# Accelerated transcranial magnetic stimulation (aTMS) to treat depression with treatment switching: study protocol of a pilot, randomized, delayed-start trial

**DOI:** 10.1186/s40814-021-00845-9

**Published:** 2021-05-05

**Authors:** Xiao Wei Tan, Edimansyah Abdin, Phern Chern Tor

**Affiliations:** 1 Department of Mood and Anxiety, Institute of Mental Health, 10 Buangkok View, Singapore, 539747 Singapore; 2 Research Division, Institute of Mental Health, Singapore, 539747 Singapore; 3 Neurostimulation Service, Institute of Mental Health, Singapore, 539747 Singapore; 4 Duke-NUS Graduate Medical School, Singapore, 169857 Singapore

## Abstract

**Background:**

Repetitive transcranial magnetic stimulation (rTMS) is a technique for stimulating brain activity using a transient magnetic field to induce an electrical current in the brain producing depolarization of focal groups of brain cells. TMS is a protocol approved by the U.S. Food and Drug Administration in routine clinical practice as a treatment for depression. A major limitation of rTMS is the large amount of time taken for a standard protocol (38 min a day for 20–30 working days). The optimal type and duration of TMS are still uncertain, as is the optimal strategy for continuing or changing the type of rTMS if there is a poor initial response.

**Objectives:**

The trial aims to assess whether a 1-week compressed course of left dorsolateral prefrontal (L DLPFC) 5 Hz accelerated rTMS (aTMS) treatment is as effective as an established 4-week course of non-accelerated rTMS and if additional 5 Hz L DLPFC aTMS treatments will be efficacious in non-responders as compared to 1 Hz right DLPFC aTMS treatment.

**Methods:**

A randomized, single-blind, delayed-start trial was planned to commence in Jan 2020. A total of 60 patients will be enrolled from the Institute of Mental Health Singapore within a 2-year period and randomized into the early or delayed-start phase of the trial. The primary outcome of the trial is the improvement of Montgomery-Asberg Depression Rating scale at the end of the active treatment phase.

**Discussion:**

If this study protocol proves to be effective, the findings of this trial will be updated to the College of Psychiatrists, Academy of Medicine Singapore, as well as published in a peer-reviewed journal to enhance local and international TMS treatment guidelines.

**Trial registration:**

ClinicalTrials.gov ID: NCT03941106

## Background

Depression confers a large burden of disease because of its early onset, high prevalence, and the profound disability involved [1]. In Singapore, the lifetime prevalence of depression is 5.8% [2]. The economic burden of depression in the Asia Pacific region [3] and Singapore [4] is high and up to 50% of indirect costs were associated with lost productivity and unemployment. Major depressive disorder (MDD) is a severe mental illness that affects major life domains, such as mental and psychosocial functioning [5, 6]. The current long-term treatment strategies remain suboptimal for patients with MDD. A large proportion of them do not respond satisfactorily to drug therapy and experience relapses over a prolonged period of their life [7, 8].

Electroconvulsive therapy (ECT) is considered to be one of the most effective treatment modalities yielding an 80–90% response rate in patients with the acute phase of depression [9] and 50–70% response rate in patients with treatment-resistant depression (TRD) [10]. Although a highly effective antidepressant treatment, the use of ECT is constrained by stigma and concern over cognitive side effects. Accordingly, great interest has developed in non-invasive brain stimulation (NIBS) therapies such as repetitive transcranial magnetic stimulation (rTMS). These NIBS techniques involve applying a weak electric current/pulse to the brain via an electromagnetic coil to depolarize neurons in targeted cortical regions (most commonly the dorsolateral prefrontal cortex, DLPFC) and thereby modulate mood.

There is now established evidence that rTMS is effective in treating depression [11–13] with approval for its clinical use in countries including the USA, Australia, Singapore, Canada, and Israel. rTMS also has an excellent safety profile, demonstrated in numerous clinical trials, including a substantial number of depressed participants [14]. Most research in rTMS has focused on the pulse frequency [15, 16] stimulation intensity [17] and duration of treatment [18, 19]. rTMS treatment of depression is cost-effective [20] compared to ECT. A local cost-effectiveness study of ECT vs*.* TMS shows that TMS is a highly cost-effective option compared to ECT for non-psychotic depression [21].

Although intermittent theta-burst stimulation (iTBS), a FDA-approved novel form of magnetic stimulation is undergoing intensive study as an alternative approach of rTMS [22–24], the original FDA approved protocol for rTMS, the left 10Hz Dorsolateral Pre-frontal cortex (10Hz L DLPFC) protocol, has a larger evidence base to date. However, there is relatively low efficacy of rTMS compared to ECT [25] and the optimal form of rTMS for patients not responding to initial rTMS modality is unclear [26]. More sessions [27], change of rTMS stimulation intensity [28], change from unilateral to bilateral rTMS treatment [29], or use iTBS to treat drug-resistant depression ([30, 31] have been studied but the results are inconclusive. Current clinical practice commonly changes rTMS modality from high-frequency Left DLFPC (L DLFPC) to low-frequency Right DLPFC (R DLPFC) [32], but there is no evidence that this is more efficacious as compared to adding more sessions of the same rTMS. Moreover, although there is a general acceptance that increased dose and duration of TMS is associated with increased efficacy of rTMS [33], there is preliminary evidence of a delayed response to rTMS [34, 35] suggesting that some patients could be treated effectively with a shorter period of treatment given every weekday, then waiting for several weeks for full clinical improvement from the treatment.

Furthermore, the original FDA-approved protocol for rTMS, which to date has the largest evidence base, is the 10Hz L DLPFC protocol. This involves one session in each working day and patients need to come for the treatment for continuously 4 weeks. For those patients with no or poor response, additional 2 weeks of treatment by rTMS will be supplemented. Therefore, a standard course of unilateral TMS is about 38 min a day for 20–30 working days, substantially limiting the capacity of TMS services to meet the treatment needs of all patients. rTMS requires a considerable time commitment from both patients and clinicians and is of limited utility for patients who do not live within convenient traveling distance from treatment centers. The time needed before treatment response in some patients also makes rTMS unsuitable for some acutely suicidal patients. Accelerating the administration of rTMS (aTMS) by administering multiple sessions of rTMS over a shorter period of time has been shown to potentially be as efficacious as standard rTMS therapy [36–43]. These studies showed that aTMS was safe and efficacious, with no significant side effects reported, with a high level of patient acceptability and significant improvements in subjects’ depression after the aTMS. Thus, aTMS can potentially offer a more cost-effective treatment for depression than standard TMS that can help alleviate the large economic burden of depression by allowing patients to return to their work more rapidly.

## Methods

### Objectives

The primary aim of this study is to test the symptomatic efficacy of aTMS trial protocol. The 2nd aim is to compare the symptomatic efficacy of the early-start aTMS treatment group and delayed-start aTMS treatment group in patients with a DSM 5 Major Depressive Episode. The exploratory aim is to test whether the non-responders to a 1-week course of aTMS will have a higher chance of response to a change in aTMS than repeated the same aTMS modality (from 5 Hz L DLPFC aTMS to 1 Hz R DLPFC aTMS).

### Hypothesis

We hypothesize that accelerated 5 Hz L DLPFC rTMS is effective for the treatment of depressive symptoms with regard to reducing the MADRS scores from baseline to end week 3 in the overall study sample.

### Design of the trial

#### Recruitment and screening

Participants will be recruited from inpatients and outpatients at the Institute of Mental Health (Singapore) by the research team psychiatrists and referrals from other psychiatrists at the Institute of Mental Health.

#### Inclusion criteria

1. Age ≥21 years.
2. DSM-5 diagnosis of current major depressive disorder.
3. Montgomery-Asberg Depression Rating Scale score of 20 or more.
4. Able to give informed consent.

#### Exclusion criteria

1. DSM-5 psychotic disorder.
2. Drug or alcohol abuse or dependence (preceding 3 months).
3. Inadequate response to ECT (current episode of depression).
4. Rapid clinical response required, e.g., high suicide risk.
5. Significant neurological disorder, which may pose increased risks with TMS, e.g., epilepsy.
6. Metal in the cranium, skull defects, pacemaker, cochlear implant, medication pump, or other electronic devices.
7. Pregnancy.

The following clinical data will be collected at baseline: age, gender, ethnicity, duration of index episode, type of depression (unipolar/bipolar, melancholic/non-melancholic), number of failed antidepressant treatments, number of previous depressive episodes, age at the first depressive episode, and family history of affective disorder will be noted. Full informed written consent will be obtained from all participants.

#### Randomization at phase 1

**Fig. 1:**
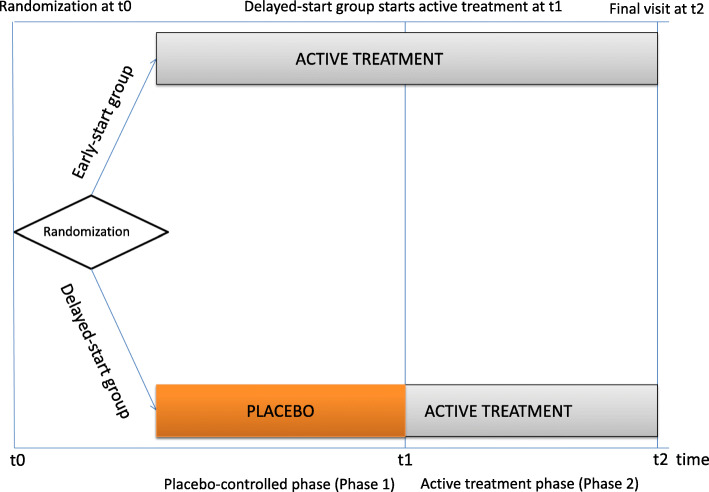
Graphical display of the delayed-start design

In phase 1, subjects were randomly assigned either to the early-start group (immediate active treatment) or the delayed-start group (no TMS treatment for one week followed by active treatment, see Fig. 1). Randomization was done using a random sequence number generator.

#### Treatment at phase 1

During the active treatment phase, all participants will receive 5 Hz aTMS over the L DLPFC, 4 treatment sessions a day (each session consisting of 3000 pulses for 12 000 pulses a day), every weekday for 1 week. We will use a Magventure X100 TMS Therapy System to conduct TMS stimulation. The first treatment session will take approximately 3 h as participants will also be familiarized with the TMS equipment/procedure and resting motor threshold (RMT) will be determined at this session. We use visual assessment of the respective dorsal interosseous muscle contraction (5 out of 10 stimulations) induced by TMS to determine RMT. Subsequent sessions are expected to last about 2 h each. Stimulation will be applied at the left prefrontal cortex (F3 according to the EEG 10/20 system). L DLPFC aTMS will be delivered at 120% of the RMT, as measured at the initial session. Each TMS train will last for 8 s and have a 12-s intertrain interval. Each session will have 75 trains. Thus, it will be taking about 25 min for each session which includes setting up time.

aTMS will be conducted in a room with facilities for managing seizures. Participants and experimenters will be offered earplugs during the sessions. Sessions will be done by staff trained and credentialed in TMS according to the Singapore College of Psychiatrists guidelines for TMS. Staff will also be trained for initial seizure management.

Patients in the delayed-start group will be treated as usual during the delayed week and will receive aTMS treatment at 1 week after recruitment. Subjects in the delayed-start group and early-start group will both be monitored with weekly outcome assessments. Subjects in the delayed start arm who still fulfill the criteria for study entry at the end of the delayed start phase will continue on to the active treatment phase of the study. No change in psychiatric medication will be allowed during this period.

#### Evaluation of clinical response at phase 1

A blinded, the trained rater will assess mood each week, from baseline to 2 weeks after treatment, using the Montgomery-Asberg Depression Rating Scale (MADRS) scale. MADRS scale is more sensitive to change in patients’ mental condition than Hamilton depression rating scale [44]. The primary outcome is response rate, which are assessed at end of week 4 and week 6. Secondary outcomes include remission rate, Clinician Global Impression –Improvement Scale (CGI-I) and the self-rated Quick Inventory of Depression (QIDS-16). Patient’s cognition will be monitored using the Montreal Cognitive Assessment Scale (MoCA) before and after the 1-week active treatment period and quality of life (QoL) measured by the Quality of Life Enjoyment and Satisfaction Questionnaire – Short Form (*Q*-*LES*-*Q*-SF), assessed at baseline and end of week 2. MoCA is a brief cognitive instrument recommended for screening for cognitive impairment (CI) in patients. Several studies have compared the discriminant abilities of the MoCA and the Mini-Mental State Examination (MMSE) for screening post-stroke CI, and most studies have demonstrated that the MoCA is superior or equivalent to the MMSE for the detection of CI after stroke. Furthermore, the MoCA has been reported to be sensitive to changes in acute temporary CI after mild stroke/TIA, whereas the MMSE is reportedly not [45]. Therefore, in the current study, we use MoCA to assess acute changes in cognitive abilities.

After aTMS treatment, all subjects will then be followed up once a week for 2 weeks and subjects who achieve remission will exit the study.

#### Randomization at phase 2

Subjects who have not remitted from the 1st active treatment will enter phase 2 treatment. The second randomization will be stratified into three groups based on whether the participant showed <25% improvement in MADRS scores, between 25% and ≤50% improvement in MADRS scores or >50% improvement in MADRS scores. After that, participants in each group will be randomized (in permuted blocks) to either L DLPFC or R DLPFC treatment. The purpose of patient stratification basing on MADRS score before randomization is a method of permuted block randomization, which is a way to randomly allocate a participant to a treatment group, while maintaining a balance across treatment groups. Each “block” has a specified number of randomly ordered treatment assignments depending on the response to the initial aTMS treatment. Randomization will be generated by study team members who are not involved in the treatment. Treatment assignment is determined by treating doctors. Patients, their caregivers, nurse clinicians, and doctors who do the treatment and outcome assessments will be blinded to the randomization.

#### Treatment at phase 2

Subjects who entered phase 2 will receive another 1 week of 5 Hz L DLPFC aTMS (same protocol as above) or aTMS consisting of right DLPFC at 1Hz, 4 treatment sessions a day (each session consisting of 3000 pulses for 12000 pulses a day), every weekday for 1 week. These patients will also be followed up with weekly assessments for 2 weeks after the end of the second round of aTMS. Therefore, participants who receive a total of 1 week of daily aTMS would have received 60000 pulses while those who received a total of 2 weeks of daily aTMS would have received 120000 pulses.

We will use the Magventure X100 to carry out low-frequency R DLPFC. 1 Hz R DLPFC aTMS will take about 4 h a day. Stimulation will be delivered at the right prefrontal cortex (F4 according to the EEG 10/20 system). R DLPFC aTMS will be delivered at 120% of the RMT, as measured at the initial session. Each session will have 300 trains which is equivalent to a consensus protocol of delivering 3000 pulses per session over the L DLPFC [46].

#### Evaluation of clinical response at phase 2

Similar to the outcome assessment during phase 1, mood improvement and subject QoL will be assessed every week during acute treatment by a trained rater (blinded to treatment allocation) using the MADRS scale until 2 weeks after the initiation of 2nd round of treatment.

In summary, the primary outcome of this study will be a mean change in the MADRS score from baseline to the end of 4 weeks after treatment initiation. The primary endpoint of the trial will be the MADRS score <11 at any time points of follow-up after initiation of rTMS treatment which indicates the patients have achieved symptoms remission hence can exit from the study.

**Fig. 2:**
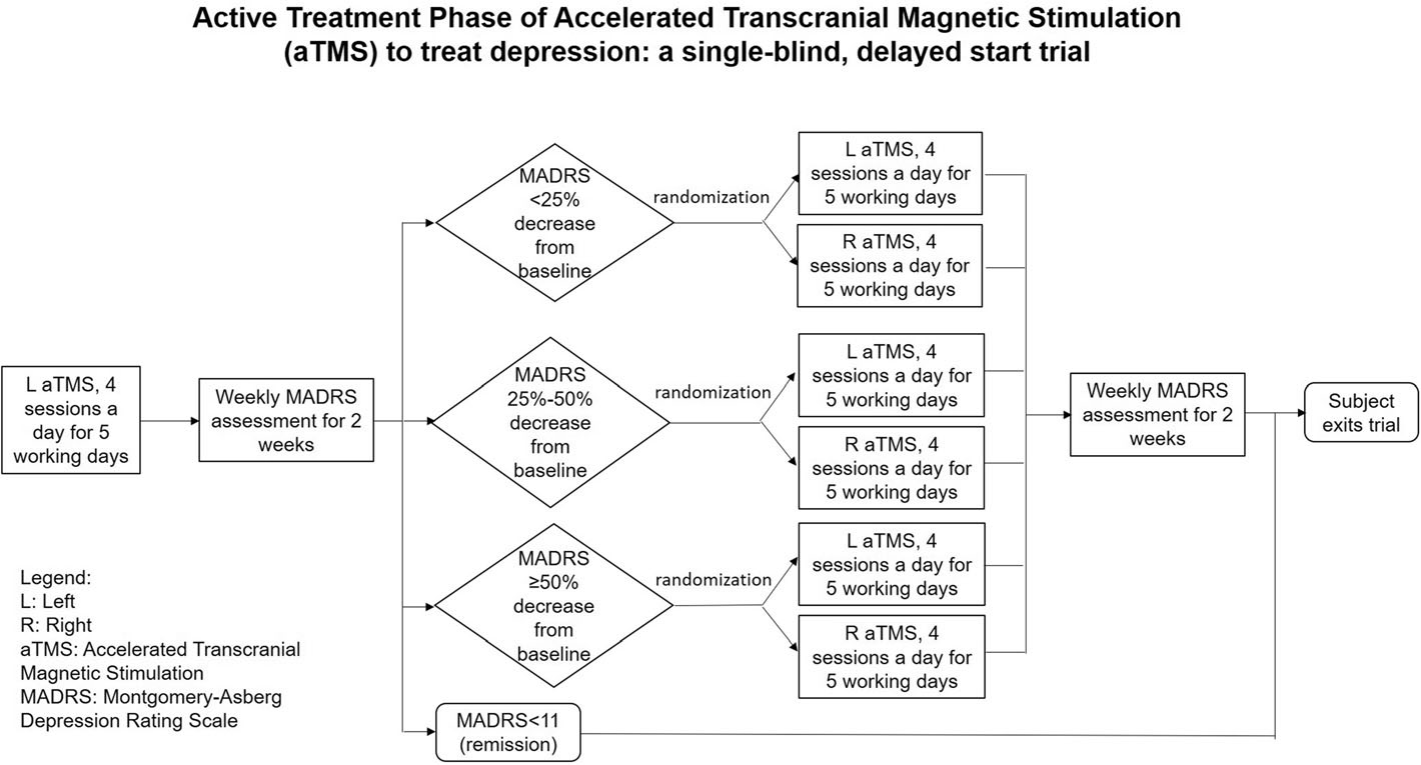
Graphical display of the active treatment phase

See Fig. 2 for the flowchart of the study design.

### Statistical analysis

Clinical, demographic characteristics and mood, cognitive, and QoL scores will be tested for any differences at baseline between the two randomized groups using *t test* or *chi-square* test wherever appropriate. The effect size of aTMS will be assessed by the proportion of response rate, which was defined as “Response: ≥50% MADRS score decrease from baseline” and “Non-response: <50% MADRS score decrease from baseline”. “Remission” is defined by score of 10 or less on MADRS score. Changes in mood and other ratings from baseline to each follow-up time point will be assessed with paired *t* test followed by repeated-measures analysis of variance (*ANOVA*)*.* Statistical tests will be two-tailed.

### Safety issues

Participants will undergo periodic observer-rated mood assessments during the study to check for any side effects or mood deterioration, including the emergence or exacerbation of psychiatric symptoms. In addition, before and after each TMS session, participants will be asked about any side effects or adverse events experienced. Responses will be documented, and spontaneous reports of the side effects will also be noted. An adverse event will be defined as any unfavorable medical change occurring that is accompanied by functional or clinical impairment. Participants who deteriorate to an unacceptable level during the trial (e.g., become acutely suicidal) will be withdrawn from the trial and appropriate clinical measures for their further treatment will be taken, including notification of their own treating doctor, referral to crisis team/community health center/public or private hospital.

1. Some participants have reported mild headache after TMS—this has not been problematic and has required mild analgesia at the most.
2. The TMS device emits a clicking noise when it delivers the stimulation. There have been reports of hearing impairment in animals (permanent) and human participants (temporary) when TMS was given without the use of earplugs. Participants and experimenters will be provided with earplugs and/or protective headphones during TMS in this study.
3. Seizures: Only a very small number of accidental seizures have been reported worldwide with TMS (*n*=11). Most of these have occurred with stimulus parameters outside current recommended safety guidelines [47]. There is no clear evidence of seizure induction with TMS in individuals who did not have an existing disorder which predisposes them to seizures. All TMS parameters used in this study are within safety guidelines. Our group has administered multiple sessions of TMS to over 26 subjects since November 2015 without accidental seizures or serious adverse effects. Safety precautions include training TMS staff in recognition and initial management of seizures. All subjects will be monitored for at least 30 min after each TMS session by trained staff nurses to monitor for possible seizures, headaches, or nausea*.*
4. Pregnancy: there are at least 2 case reports of the safe use of repeated sessions of TMS (given in the first, second, and/or third trimester) to treat depression in pregnant women [48, 49]. Further, there are no theoretical reasons to suggest that TMS would pose a specific risk to the pregnant woman or fetus [49]. However, as the aim of the study is not to assess the safety of TMS in pregnancy, and as the risks of TMS in pregnancy are still relatively unknown, pregnant women will be excluded from the study.
5. Mood switching: there are 6 case reports of TMS causing a mood switch into mania in participants with bipolar disorder [50]. The risk is relatively low, given the number of participants who have received TMS. Participants with unipolar depression who have had previous mood switching induced by an antidepressant treatment or with bipolar disorder will be advised that they may be at increased risk of mood switching with TMS and will be asked to consult their own doctor about the need for mood-stabilizing medication. There is no evidence that TMS is more likely to induce a manic switch than another course of antidepressant medication, i.e., the main alternative treatment for participants recruited into this study. Participants will be carefully monitored for mood changes suggestive of mood switching and if this occurs, will discontinue the trial and receive appropriate clinical treatment.

### Sample size calculation

Based on our center’s TMS results to date, patients start TMS treatment with an average MADRS score of 25.4 (SD 7.3), which decreases significantly to 18.4 (SD 9.9) after 2 weeks of treatment with standard rTMS and to 14.1 (SD 10.7) after 4 weeks of treatment. Participants enrolled in the study would be expected to have similar improvements in MADRS with aTMS treatment.

This study is powered to test the primary aim, i.e., a significant reduction in MADRS scores from baseline to end week 3, in the overall study sample. Based on the MADRS scores cited above for baseline (25.4 ± 7.3) and end week 4 (14.1 ± 10.7) time points, detecting a similar significant change after a course of aTMS would require a sample of 12 participants (alpha 0.5, 95% power). However, given the lack of data on the efficacy of aTMS in an Asian population, and to give a better estimate of response and remission rates, we anticipated that 60% of those recruited will drop out before the end of the trial with an unequal allocation ratio of 2:1, we thus aim to recruit 40 subjects in the delayed start arm and 20 subjects in the immediate start arm for a total of 60 subjects.

No prior data exist on the comparative effects of delayed response with immediate treatment of aTMS (or TMS), we will also explore the aim that non-responders to a 1-week course of aTMS will have a higher chance of response to a change in aTMS from L 5 Hz DLPFC TMS to R 1 Hz DLPFC for depression. This study will produce pilot data for 2nd and exploratory aim, informing the power analysis for a future clinical trial if results reject a null hypothesis.

## Discussion

This research proposal both addresses important practical and scientific considerations in depression, a major international [1] and local [2] public health challenge that is a leading cause of years lived with disability [51]. TMS has the potential to be a significant treatment for depression if it can be efficiently delivered to patients in need. The ability to compress TMS via aTMS could multiply the practical applicability to many more patients in the community with depression who lack the time and resources for the standard 38-min daily session over 4 weeks.

In addition, there is the scientific question of how to optimize TMS treatment after initial poor response which has been poorly investigated thus far. There is insufficient evidence on the optimal duration of TMS treatment for patients with good response. The results of this study will be of significant interest to the wider research community looking into the optimization of TMS treatment in depression.

Moreover, the results of this study have significant potential to affect local and international routine clinical practice of TMS for depression. Potentially, all restructured hospitals and more private psychiatrists could be offering TMS treatment for depression and having evidence that it can be accelerated will significantly increase adoption of this novel treatment for depression. It will also improve patient care as patients can potentially respond in 1–2 weeks rather than the 4–6 weeks of standard TMS care today. This increased speed of response will not only relieve suffering and distress but improve patient productivity by reducing time spent on treatment and accelerate their return to work or societal duties.

### Strengths and limitations

This trial has several strengths. The delayed start phase will decrease the likelihood of a significant placebo response by subjects who respond to increased attention in a clinical trial. The psychological response elicited by placebos is very specific depending on various factors such as the patient’s expectations, feelings, beliefs, volition, and hope for improvement [52, 53]. Growing placebo response is an important issue of antidepressant therapy for clinical trials involving patients with MDD [54–57]. A recent meta-analysis summarized from 61 large randomized clinical trials (RCTs) suggesting that there is a large placebo response for rTMS trial regardless of the modality of intervention [58]. In our trial, we designed a 1-week delay of treatment to possibly mitigate the placebo effect of active treatment by rTMS. However, it remains unclear whether this delayed-start phase may also reduce the anti-depressant effect of active treatment as some opinions exist that placebo response may be a component of therapeutic response to rTMS in MDD [58]. Patients will receive treatment as usual during the delayed week without coming to rTMS clinic. During the delayed phase, there is still weekly MADRS assessment and this will help patients maintain good adherence with the trial as the patients will keep informed about the study and establish a positive relationship and engagement with clinical staff.

We proposed a 2-week follow-up period after patients have been treated with compact 20 sessions of rTMS. Several open-label large-scale RCTs have shown that some patients with standard treatment of rTMS experienced a delayed response and required 4–6 weeks of stimulation before showing adequate response [12, 59–61]. Similarly, although the accelerated form of rTMS treatment with multiple sessions conducted daily may induce a rapid anti-depressant effect, there are still possibilities that some patients remain non-responding or poor-responding immediately after completion of prescribed sessions of rTMS. Therefore, 2 weeks follow-up period was proposed in this study to allow us to identify this specific group of patients with delayed responses.

The proposed study is an open-label single-blinded study and will have the limitations of a lack of a control group in which patients will receive sham-operated magnetic stimulation of the brain and a control group to minimize the expectancy bias by raters. TMS is now a standard treatment for depression [62, 63]. Depression is a severe illness that could result not only in significant distress and economic burden for patients and society, but even suicide. It would be difficult to argue for a placebo-controlled arm in a pilot study of TMS with the availability of effective treatments for depression. In addition, we have both international [29, 64–66] and local evidence of the effectiveness of TMS in actual clinical settings to be able to meaningfully interpret the data from this open-label single-blinded study of TMS in treating depression. Nevertheless, proposing various groups of control without proper treatment of rTMS is unrealistic, particularly with the current recruitment capacity of a single research institute. Future large trial with multi-site collaboration may help to solve this issue.

## Conclusions

In summary, this trial is expected to clarify the clinical advantages of compressed TMS treatment vs. the standard treatment protocol of TMS. If this trial reveals the appropriate protocol of aTMS, this result can be published to enhance local and international TMS treatment guidelines.

## Data Availability

The datasets generated and/or analyzed during the current study are not publicly available due to restrictions from the Institutional Research Review Committee, Institute of Mental Health and the National Healthcare Group Domain Specific Review Board, Singapore, but are available from the corresponding author on reasonable request.
